# Prickle1 regulates neurite outgrowth of apical spiral ganglion neurons but not hair cell polarity in the murine cochlea

**DOI:** 10.1371/journal.pone.0183773

**Published:** 2017-08-24

**Authors:** Tian Yang, Jennifer Kersigo, Shu Wu, Bernd Fritzsch, Alexander G. Bassuk

**Affiliations:** 1 Department of Biology, University of Iowa, Iowa City, Iowa, United States of America; 2 Department of Pediatrics, University of Iowa, Iowa City, Iowa, United States of America; University of South Florida, UNITED STATES

## Abstract

In the mammalian organ of Corti (OC), the stereocilia on the apical surface of hair cells (HCs) are uniformly organized in a neural to abneural axis (or medial-laterally). This organization is regulated by planar cell polarity (PCP) signaling. Mutations of PCP genes, such as *Vangl2*, *Dvl1/2*, *Celsr1*, and *Fzd3/6*, affect the formation of HC orientation to varying degrees. *Prickle1* is a PCP signaling gene that belongs to the prickle / espinas / testin family. Prickle1 protein is shown to be asymmetrically localized in the HCs of the OC, and this asymmetric localization is associated with loss of PCP in *Smurf* mutants, implying that Prickle1 is involved in HC PCP development in the OC. A follow-up study found no PCP polarity defects after loss of Prickle1 (*Prickle1*^*-/-*^) in the cochlea. We show here strong *Prickle1* mRNA expression in the spiral ganglion by *in situ* hybridization and β-Gal staining, and weak expression in the OC by β-Gal staining. Consistent with this limited expression in the OC, cochlear HC PCP is unaffected in either *Prickle1*^*C251X/C251X*^ mice or *Prickle1*^*f/f*^*; Pax2-cre* conditional null mice. Meanwhile, type II afferents of apical spiral ganglion neurons (SGN) innervating outer hair cells (OHC) have unusual neurite growth. In addition, afferents from the apex show unusual collaterals in the cochlear nuclei that overlap with basal turn afferents. Our findings argue against the role of Prickle1 in regulating hair cell polarity in the cochlea. Instead, Prickle1 regulates the polarity-related growth of distal and central processes of apical SGNs.

## Introduction

The organ of Corti (OC), the mammalian sensory organ for hearing, is located within the cochlea, and is the most sophisticated cellular assembly of the mammalian body [1]. In addition to several distinct types of supporting cells, the OC has two types of sensory hair cells (HCs) whose apical surfaces are topped with hair-like processes, known as stereocilia and a transient kinocilium [2]. The stereocilia form a V-shape pattern on the apical surface of HCs with the kinocilium located at the tip of the V, pointing toward the abneural edge of the cochlea. This polarity is essential for HCs to precisely translate mechanical stimulation from sound to electric signal [3, 4].

Planar cell polarity (PCP) signaling is crucial for the formation of HC PCP in the cochlea. Its core members include Vang-like 1/2 (Vangl1/2), Frizzled class receptor 3/6 (Fzd3/6), Dishevelled segment polarity protein 1/2/3 (Dvl1/2/3), and Prickle planar cell polarity protein 1/2/3/4 (Prickle1/2/3/4), among others [4, 5]. Some of these core PCP proteins are localized asymmetrically at the cell membrane during PCP development: Vangl1/2 are expressed in HC-SC (supporting cell) boundary medial to HCs, mostly in the supporting cells [6–8]; Fzd3/6 are expressed in the medial side of HCs [9]; Dvl1/2/3 are expressed in the lateral side of HCs [10, 11]. Disruption of one protein normally affects distribution of other core PCP proteins. For instance, in *Vangl2 Looptail (Lp)* mutants, the asymmetric distribution of Fzd3/6 and Prickle2 is lost [6, 8, 9]. Single mutation of two PCP genes, *Vangl2* [12] and *Celsr1* [13], leads to misoriented HCs. Probably due to redundancy of PCP gene families, single loss of other PCP genes does not cause PCP defects in the cochlea. Instead, it requires combined loss of multiple PCP genes, such as *Dvl1; Dvl2* double mutants, *Fzd3; Fzd6* double mutants, and *Vangl1*^*Gt/+*^*; Vangl2*^*Lp/+*^ mutants, to cause misorientation of hair cells [6, 9, 10, 13–20]. In addition, there are genetic interactions between PCP genes. For instance, *Dvl3*^*-/-*^*; Vangl2*^*Lp/+*^ mice have severely misoriented cochlear HCs while *Dvl3*^*-/-*^ only have mild defects and no detectable defects in *Vangl2*^*Lp/+*^ cochlea [11].

How PCP signaling and other signaling paradigms contribute to the asymmetric patterning of HCs is not completely understood. It is proposed that PCP signaling synchronizes HC polarity across epithelia, whereas HC polarity requires kinocilia [21] and various proteins (see review [4]) to move the kinocilia and to regulate the height and distribution of stereocilia in properly polarized hair cells.

Prickle1 is a core member of the PCP signaling paradigm. Prickle1 protein has a PET (Prickle, Espinas and Testin) domain and three LIM (Lin11, Isl-1 and Mec3) domains, both of which are protein-protein interaction domains [22–25]. At the C-terminus, it has nuclear localization signals, N-glycosylation sites, a prenylation motif, and cAMP-dependent protein kinase A sites, all of which are necessary for the protein to trans-localize to the nucleus [22, 26]. Numerous studies have suggested a role for Prickle1 in the formation of HC PCP. First, Prickle1 protein was found to be asymmetrically localized on the medial side of the cochlear HCs and this asymmetric localization is impaired when HC polarity is lost in *Smurf* mutants [27]. Second, Prickle2, a homolog of Pricklel1, is asymmetrically localized in the inner pillar cells and non-sensory cells flanking the organ of Corti, and the localization is disrupted in *Vangl2* mutants [8]. In addition, Testin, a protein that shares a PET and 3 LIM domains with Prickle1[28, 29], plays a role in the HC PCP in the OC [30]. Despite these findings, HC PCP is not affected by loss of Prickle1 protein (*Prickle1*^*-/-*^) [31]. Rather, the actin bundle of stereocilia is malformed [31]. Because another *Prickle1* mutant (*Prickle1*^*LacZ/LacZ*^ [32]) is early lethal, while the *Prickel1*^*-/-*^ mutants used in this HC polarity study survive until post-natal day 2 (P2) [31], we reasoned the lack of polarity defects in this specific *Prickle1*^*-/-*^ mutant could be due to incomplete knockout of the protein in this line. To analyze if Prickle1 regulates HC PCP in the cochlea, we analyzed two additional *Prickle1* mutant mouse lines: *Prickle1C251X* and *Prickle1*^*f/f*^*; Pax2-cre*. *Prickle1C251X* introduces a premature stop codon in the third LIM domain of the protein, and thus takes out the third LIM domain, N-glycosylation motifs, protein kinase A phosphorylation sites, nuclear localization signals and a farnesylation motif [22, 23, 33]. This mutation causes a truncated protein which is possibly a dominant-negative mutation [26], if there is any protein made. The homozygous mutant mice die around birth with aberrations in limb and palate growth and abnormal migration of facial branchial motor neurons [34–36]. *Prickle1*^*f/f*^*; Pax2-cre* mice specifically knocks out Prickle1 expression in the inner ear by taking out the start codon carrying exon 2 of *Prickle1* using *Pax2-cre* [31]. Therefore, there should be no Prickle1 protein made in the inner ear, and we can analyze the function of Prickle1 in post-hearing mice. Consistent with the previous report on the *Prickle1*^*-/-*^ mutant, we did not observe obvious HC PCP defects in the OC in either mouse line. In order to further validate our findings, we checked mRNA expression by whole mount *in situ* hybridization in the cochlea at the time when hair cell polarity develops [2]. Instead of HCs, we could only detect profound expression of *Prickle1* in the spiral ganglion, most likely in the spiral ganglion neurons. Consistent with this expression, we found that neurite growth to the apical OC and to the cochlear nuclei is affected in these mutants. Our study is the first to show that PCP signaling is involved in distal and central neurite outgrowth of the ear.

## Materials & methods

### Mice

All animal procedures were approved by University of Iowa IACUC (ACURF 0804066) and (ACURF 1109204).

The *Prickle Cys251X* (*Prickle1C251X*) mice have been previously described [34–37]. Noon on the day of vaginal plug visualization was deemed as E0.5. The PCR primers used were: P1 5’-TTTGTGCTCAGAGCCAGTG-3’, P2 5’-CAAGCGTTAAAGAAGCAAGG-3’. The PCR product was 378 bp, which was then sent to sequencing to verify the mutation. The *Prickle1*^*C251X/C251X*^ mice were born at the expected Mendelian ratio. Littermate *Prickle1*^*+/+*^ mice were used as controls unless noted otherwise.

*Prickle1LacZ* mutant mice was described previously [32, 37].

We bred mice carrying the *Pax2-cre* transgene [38] with mice carrying floxed *Prickle1* [31]. Prickle1 LoxP was genotyped using primers as in described [31] (forward: 5’-AGG AAA TCT GGG GGA CTG AG-3’ and reverse 5’-GCC ACT CAG GCA ATT AGG AA-3’). Pax2-cre was genotyped using Cre-specific primers (forward: 5′-GAA CCT GAT GGA CAT GTT CAG G-3′ and reverse: 5′-AGT GCG TTC GAA CGC TAG AGC CTG T -3′), which produced a 249 bp product. *Prickle*^*f/+*^ mice, *Prickle1*^*f/f*^ mice, and *Prickle1*^*f/+*^*; Pax2-cre* mice were used as controls.

All mice were intracardially perfused with 4% paraformaldehyde (PFA) in 1X phosphate buffer (PBS) following Avertin anesthesia (1.25% of 2.2.2-tribromoethanol at a dose of 0.025 ml/g of body weight). Heads were isolated and fixed in 4% PFA at least for 24 hours for *in situ* hybridization and immunohistochemistry experiments. The ears from animals older than P7 were decalcified in 10% EDTA in 0.4% PFA before being dissected for further processing. For beta-Galactosidase (β-Gal) staining experiment, heads were only fixed in 0.4% PFA for half an hour after perfusion. Decalcification was performed after β-Gal staining was performed.

### *In situ* hybridization

*Prickle1*^*+/+*^ or C57BL/6 mice were used to analyze gene expression. The *Prickle1* probe [39] for *in situ* hybridization was generated by *in vitro* transcription from the plasmid and then labeled with digoxigenin. Dissected cochleae were digested with 20 mg/ml of Proteinase K (Ambion, Austin, TX, USA) for 20 min, and then hybridized overnight at 60°C to the riboprobe in hybridization solution containing 50% (v/v) formamide, 50% (v/v) saline sodium citrate and 6% (w/v) dextran sulfate. After washing off the unbound probe, the samples were incubated overnight with an anti-digoxigenin antibody conjugated with alkaline phosphatase (Roche Diagnostics GmbH, Mannheim, Germany). The samples were reacted with nitroblue phosphate/5-bromo, 4-chloro, 3-indolil phosphate (BM purple substrate, Roche Diagnostics GmbH, Mannheim, Germany), which changed the color to purple by alkaline phosphatase. The reaction was stopped by 4% PFA. Samples were then mounted in glycerol and viewed in a Leica M205 FA microscope. Images were captured with Nikon E800 compound microscope using Metamorph software. At least three mice were used for either *Prickle1* or *Vangl2* at any of the stages.

### X-gal staining

After the mice were intracadially perfused, the inner ears were dissected out and briefly fixed with 0.4% PFA in 1X PBS for 30 min. After rinsing in wash solution (0.1 M sodium phosphate, 0.1% deoxycholic acid, 0.2% NP40, 2 mM magnesium chloride), the inner ears were stained in wash solution containing 1 mg/ml X-gal (5-bromo-4-chloro-3-indolyl-β-D-galactoside). The stained inner ears were decalcified if needed, dissected, mounted in glycerol, and imaged with Nikon E800 compound microscope using Metamorph software.

### Immunochemistry

The ears were blocked with 5% normal goat serum in PBS containing 0.5% Triton X-100 for 1 hour. Then the primary antibodies for Myo7a (1:200; Proteus Biosciences, 25–6790), and β-tubulin (1:800; Sigma, T7451), or conjugated phalloidin-568 (1:40; Molecular Probes), were added and incubated for overnight at 4°C. After several washes with PBS, corresponding secondary antibodies (1:500; Alexa fluor 647 and 488; Molecular Probes) were added and incubated for 1 hour at room temperature. The ears were washed with PBS and mounted in glycerol and images were taken with a Leica TCS SP5 confocal microscope.

### Lipophilic dye tracing

Heads from six P0 *Prickle1*^*C251X/C251X*^ mutants and their littermate controls were split along the midline. Half of the head was used for tracing of afferents to the OC, and the other half of the head was used to trace afferents (and efferents) to cochlear nuclei.

To trace afferents to the OC, six cochleae of the mutant and six controls were micro-dissected, split into base and apex. Small slivers of NeuroVue dye wafers were inserted into the spiral ganglion [40]. Cochleae were incubated over night at 60°C. After removing the dyes, cochleae were mounted, and fibers growing to OHCs were imaged using a Leica SP5 with 40x 1.3 NA lens. Image stacks were collected at 1 μm interval. Afferents growing toward the apex or branches were analyzed where limited labeling allowed tracing of single fibers.

To trace afferents (and efferents) to cochlear nuclei, two different colored lipophilic dye wafer slips [41] were inserted in the base and apex. Samples were incubated at 60°C for three days as previously described [42]. Brain halves, cochleae and cochlear nerve were prepared and imaged using a Leica SP5 confocal microscope to reveal accuracy of applications of dye, trajectory of afferents (and efferents) in the cochlear and vestibular nerves and the central target nuclei.

### Scanning electron microscopy (SEM)

Dissected (and decalcified) ears were post-fixed in 2.5% glutaraldehyde overnight, followed by rinsing in 0.1 M phosphate buffer (pH 7.4) and secondary fixation with 1% osmium tetroxide in 0.1 M phosphate buffer. After washing several times in distilled water to remove all ions, the cochleae were dehydrated in a graded ethanol series and further dehydrated using a critical point dryer. The ears were then mounted on stubs with carbon tape and coated with gold/palladium using a K550 Emitech sputter coater at 10 mA for three minutes. Samples were viewed with a Hitachi S-4800 SEM using a 10 μA emission current.

## Results

### Prickle1 is expressed in the spiral ganglion of the cochlea

**Fig 1:**
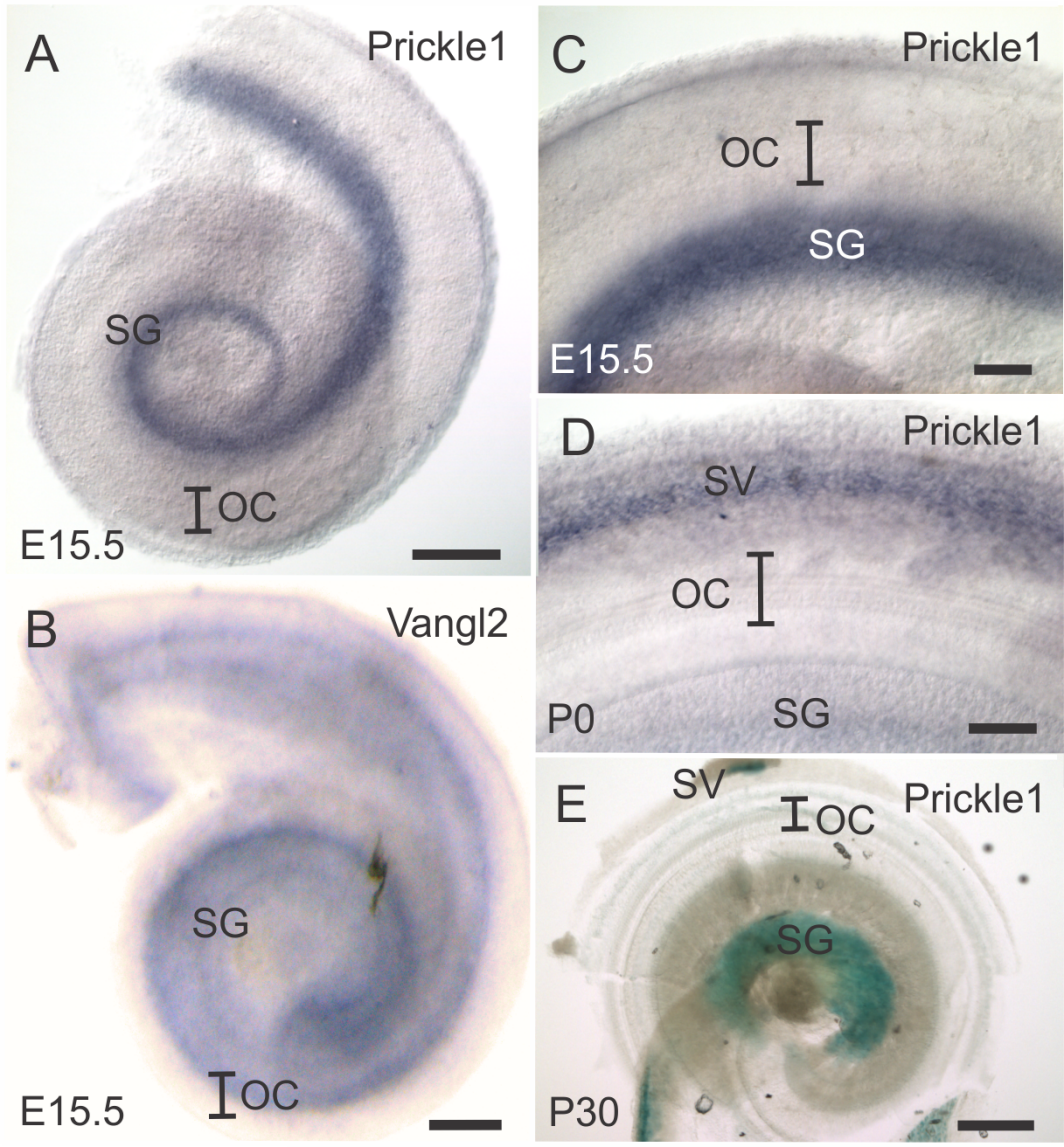
*Prickle1* is expressed in the spiral ganglion but not the organ of Corti by *in situ* hybridization during development. (A-D) *Prickle1* and *Vangl2* mRNA expression was analyzed by whole mount *in situ* hybridization in wild-type cochleae. (A-B) An overview of the cochlea showing *Prickle1* (A) and *Vangl2* (B) expression at E15.5. (C-D) A higher magnification of the cochlea showing *Prickle1* mRNA at E15.5 (C) and P0 (D). (E) β-gal staining was performed in *Prickle1*^*LacZ/+*^ cochlea to analyze Prickle1 expression at P30. Only the apex is shown. SG, spiral ganglion; SV, stria vascularis; OC, organ of Corti. Scale bar, A, B and E, 200 μm; C and D, 100 μm.

To understand the possible effects of Prickle1 in the inner ear, we first used whole mount mRNA *in situ* hybridization to analyze the expression of *Prickle1* in the cochlea when the HC PCP is developing. Using *in situ* hybridization avoids false positive or incomplete labeling with an antibody, and allows for detection of all Prickle1 isoforms. Unexpectedly, at E15.5 (Fig 1A and 1C) and P0 (Fig 1D), no *Prickle1* signal was detected in the organ of Corti (OC). Instead, *Prickle1* was expressed in the spiral ganglion (SG). This contrasted to *Vangl2*, whose signal was strong in the OC at E15.5 (Fig 1B). To confirm the expression of *Prickle1*, we performed β-gal staining in P1, P6 (S1 Fig) and P30 (Fig 1E) cochleae in *Prickle1*^*LacZ/+*^ mice [32]. The β-gal signal revealed strong expression of *Prickle1* in the SG but very weak expression in the OC. We also detected *Prickle1* expression in the stria vascularis (SV) (Fig 1D and 1E).

### Prickle1 regulates neurite growth of the type II spiral ganglion neurons (SGNs) in the apex

**Fig 2:**
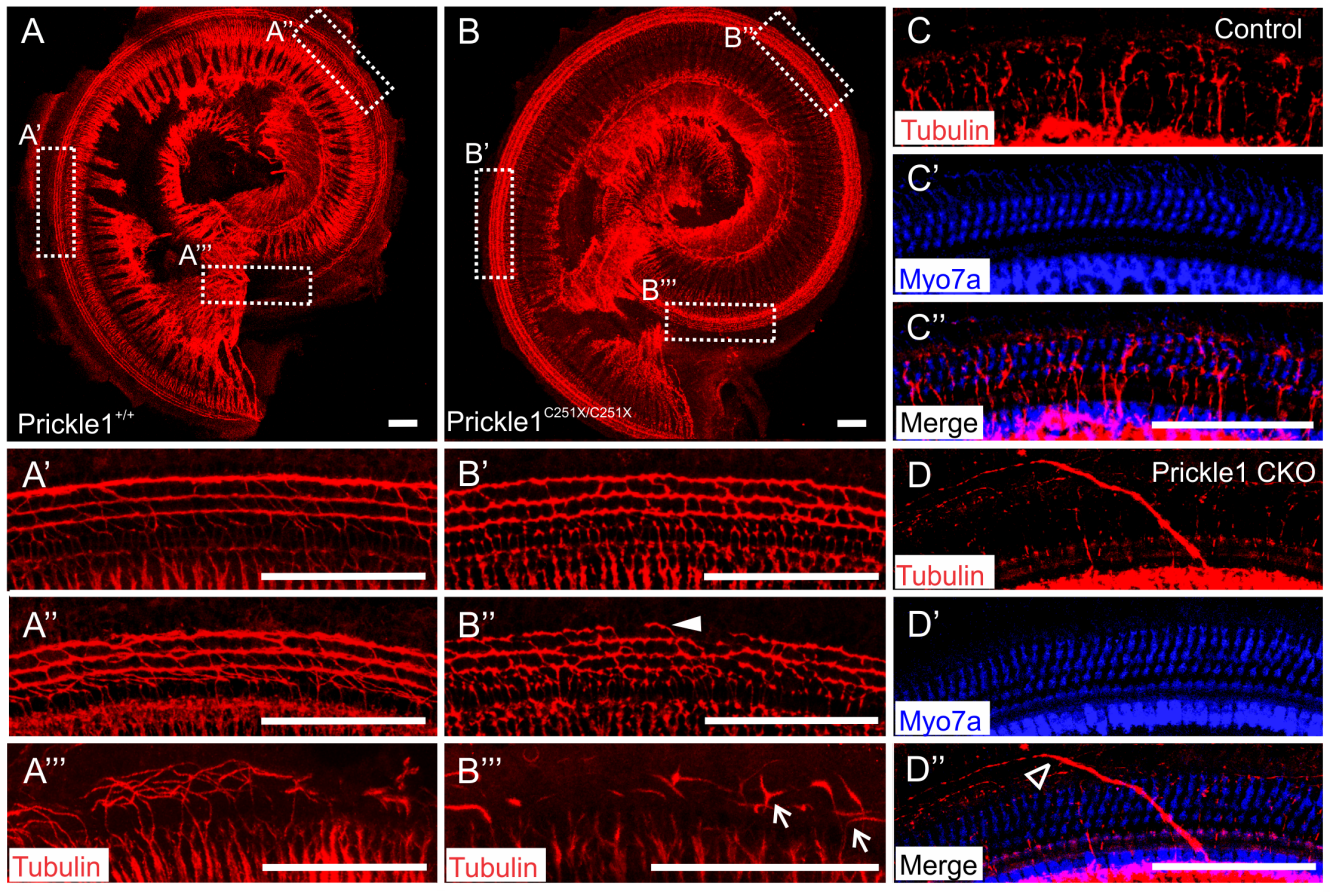
Prickle1 mutation impairs type II fiber outgrowth. (A-B”‘) The outgrowth of type II SGN afferents was analyzed by immunocytochemistry in *Prickle1C251X* mutants at P0. Cochleae were immuno-labeled with antibodies against β-tubulin. Overview of the *Prickle1*^*+/+*^ (A) and *Prickle1*^*C251X/C251X*^ (B) cochleae, and the type II afferents from the corresponding basal (A’ and B’), middle (A” and B”), and apical (A”’ and B”’) turns are shown. (C-D”) The outgrowth of type II SGN afferents analyzed by immunocytochemistry in *Prickle1*^*f/f*^*; Pax2-cre* mice. Cochleae were immune-labeled with antibodies against Myo7a and β-tubulin, and the outgrowth of type II SGN afferents are shown in control mice (C-C”) and in *Prickle1*^*f/f*^*; Pax2-cre* mutants (D-D”) at P33. Arrow, type II afferents turning towards the apex; filled triangle, type II afferents still developing; empty triangle, fibers that grew past the outer hair cells. Scale bar, 100 μm.

In the cochlea, type I SGNs innervate the inner hair cells while type II SGNs innervate the outer hair cells [43]. The type II afferents form three rows of fibers associated with the three rows of outer hair cells. All type II fibers turn toward the base of the OC. This development begins at the base at around E16.5 and progresses towards the apex. Since Prickle1 regulates neuron morphogenesis and function in other developing systems [26, 36, 39, 44, 45], we reasoned that Prickle1 might regulate the outgrowth of the type II afferents. Therefore, we labeled the neurites with anti-β-tubulin antibodies [46]. At P0 (Fig 2A), there were three rows of fibers at the basal (Fig 2A’) and middle (Fig 2A”) turns in the *Prickle1*^*+/+*^ cochleae, while the neurites were still growing in the apical turn (Fig 2A”’). In *Prickle1*^*C251X/C251X*^ mutants (Fig 2B), even though neurites formed three rows of fibers at the basal turn (Fig 2B’), neurites were not fully developed in the middle turn (Fig 2B” filled triangle). In addition, we observed some type II afferents turn towards the apex instead of the base (Fig 2B”’, arrow).

*Prickle1*^*C251X/C251X*^ mice die around birth. To analyze the type II fiber growth in older mice, we analyzed 1-month old *Prickle1*^*f/f*^*; Pax2-cre* mice, when the outgrowth of type II afferents is complete. We labeled the cochlea with antibodies against β–tubulin and Myo7a to label the neurites and hair cells. Compared with control mice (Fig 2C–2C”), we observed occasional abnormal trajectories of type II afferents projected past hair cells (Fig 2D, empty triangle).

**Fig 3:**
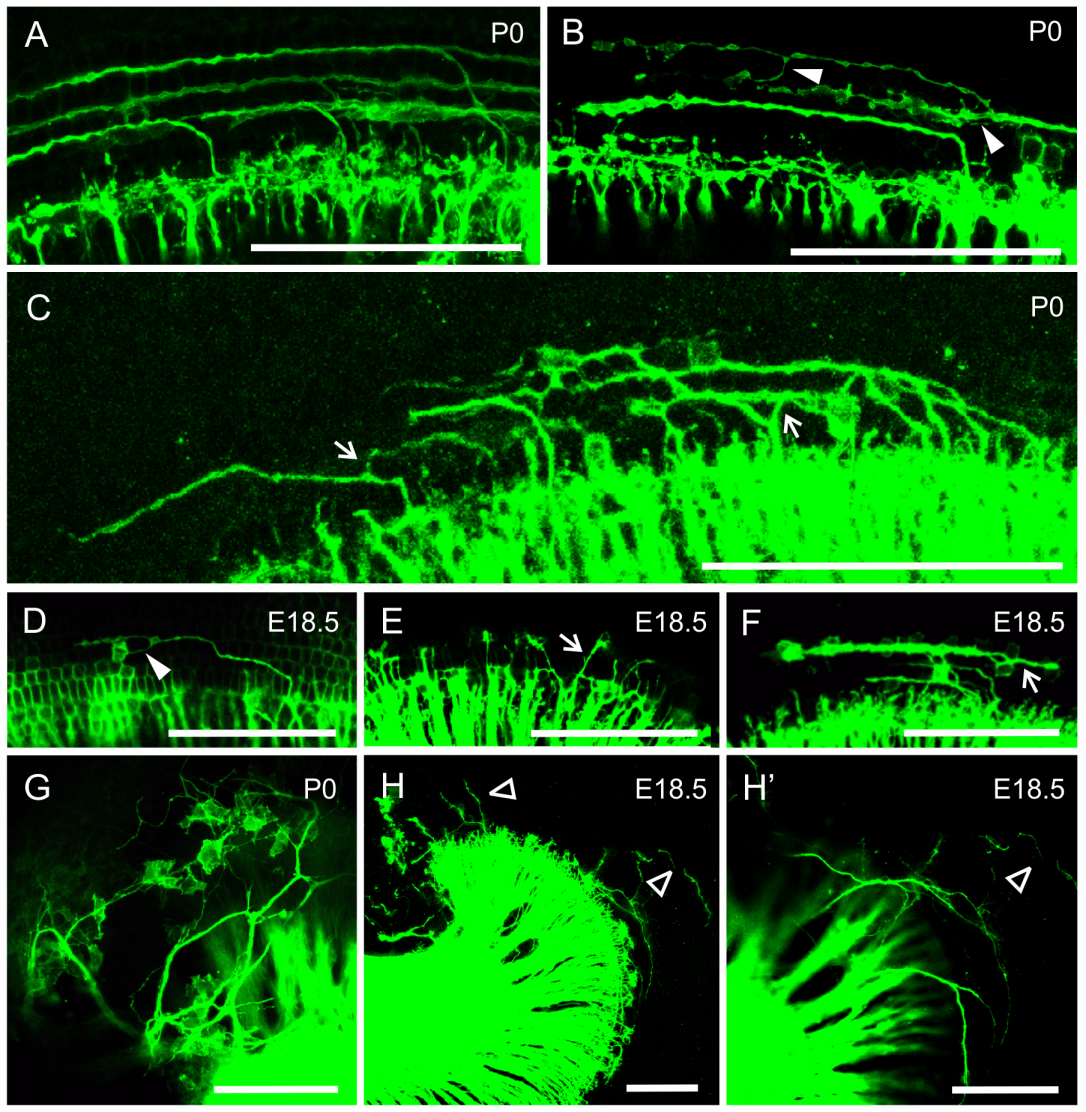
Loss of Prickle1 leads to aberrant afferent outgrowth in the apical cochlea. A select population of type II fibers was labeled by dye tracing in *Prickle1*^*C251X/C251X*^ mutants and their littermate controls at E18.5 and P0. (A) In the base, both control and *Prickle1*^*C251X/C251X*^ mutants formed three rows of type II fibers growing towards the base. (B-F) In the apex of the *Prickle1*^*C251X/C251X*^ mutant cochlea, outgrowth of some type II afferents was disrupted. (G and H’) Some afferents were not in the same focal plane as the radial fibers growing towards the hair cells (HCs). (H’) A higher magnification view of (H). Filled triangle, fibers that branched; arrow, fibers growing toward the apex; empty triangle, fibers that grew past HCs.

We further analyzed the aberrant outgrowth of type II afferents with lipophilic dye sparse tracing. In the base of both control and *Prickle1*^*C251X/C251X*^ OC, the type II afferents grew towards the base and formed three rows of fibers as they were growing out before E18.5 [47, 48] (Fig 3A). However, in the apex of *Prickle1*^*C251X/C251X*^ OC where we could follow single afferents, we observed that about 20% of type II fibers that we scored (n = 80 single fibers) branched multiple times to innervate more than one row of OHCs (Fig 3B–3F, filled triangle). We also observed about 10% of type II fibers analyzed (n = 80) grew towards the apex (Fig 3B–3F, arrow). Interestingly, in the majority of cases, the afferents turning towards the apex were also branches of afferents (Fig 3C compared with 3E). To our knowledge, type II afferents branching to innervate different rows of OHCs has not been described previously [48]. In addition, some of these aberrant afferents failed to innervate hair cells (Fig 3G–3H’): while some afferents extended past hair cells towards the lateral wall (Fig 3H and 3H’, empty triangle), some afferents separated from the radial fibers to project at the level below the rest of the radial fibers and HCs (Fig 3G and 3H’), extending past the basilar membrane to the lateral wall as previously reported in neurotrophin [49] and Schwann cell mutants [50]. These data show that some type II afferents make non-stereotyped branches that fail to project to HCs in *Prickle1C251X* mutants. Together, our results show Prickle1 plays a role in the outgrowth of distal type II SGN afferents, and *Prickle1C251X* mutants have a more severe phenotype than *Prickle1CKO* mutants.

### Apical spiral ganglion afferents expand their cochlear nucleus projection area

**Fig 4:**
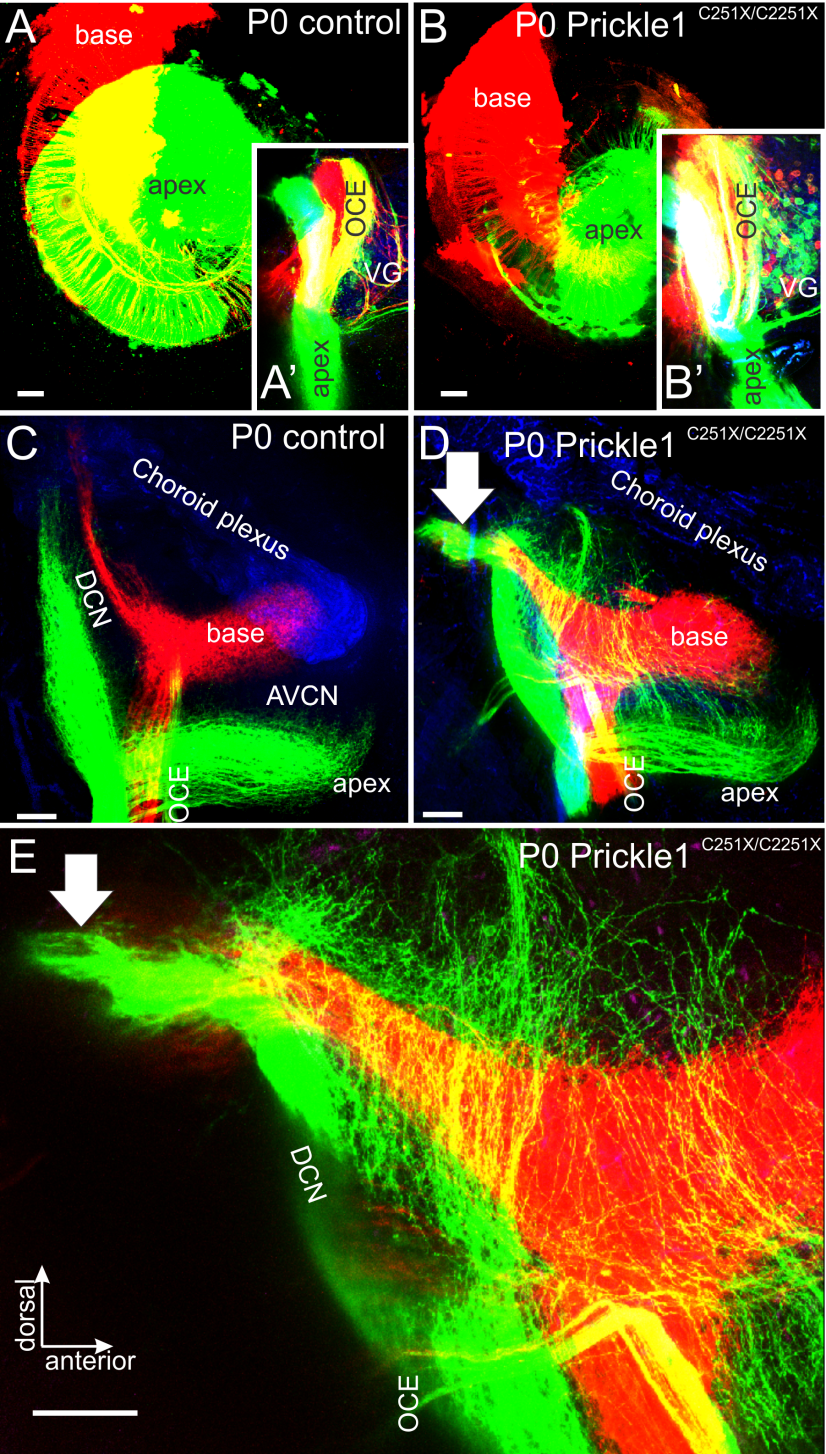
Central projections from apical afferents are expanded in the cochlear nuclei in *Prickle1*^*C251X/C251X*^ mutants. Different colors of lipophilic dyes were applied to apex and the base of the cochlear (A, B), and their central projection were analyzed (A’, B’, C-E). (A and B) Overview of the cochlea showing the application of red dye to the base and green dye to the apex in control (A) and *Prickle1*^*C251X/C251X*^ mutant (B). After 3 days of diffusion, there was partial overlap of the dye. (A’ and B’) Selective bundles of afferents and olivocochlear efferents (OCE) passed along the vestibular ganglion (VG). Only in *Prickle1*^*C251X/C251X*^ mutant (B’), OCE separated into several bundles. In addition, several vestibular ganglion neurons (VG) were labeled. (C-E) Projections to the cochlear nucleus of the control (C) and the *Prickle1*^*C251X/C251X*^ mutant (D, E). (C) In controls, afferent bundles from the apex and the base segregated and formed distinct fascicles. (D, E) In the mutant, although afferents from the base projected normally to the dorsal cochlear nucleus complex DCN, afferents from the apex formed collaterals that spread out throughout the DCN and the anterior-ventral cochlear nuclei (AVCN). (E) Higher magnification of the DCN of (D), showing details of apical afferents passing basal turn afferents to branch in the most dorsal aspect of the cochlear nucleus complex. Arrow, afferents innervating vestibular nuclei. Scale bars, 100 μm.

In addition to stereotypical topographical distal connections to the cochlear HCs, SGNs also project topographically to cochlear nuclei at the embryonic stage (Fig 4A and 4C) [47], which are the bases for tonotopic (frequency) specific hearing [51, 52]. To analyze if the central projection of SGNs was regulated by Prickle1, we injected different colored dyes into the apex and the base of the cochlea (Fig 4A and 4B), and analyzed the central projection in the cochlear nuclei (Fig 4). Our results revealed that projections to cochlear nuclei were not as segregated in *Prickle1*^*C251X/C251X*^ mutant mice as in control animals at various levels. First, the olivocochlear efferents (OCE) of the *Prickle1*^*C251X/C251X*^ mutant separated into several bundles (Fig 4B’), rather than a compact nerve (Fig 4A’). Second, more vestibular ganglion (VG) neurons were labeled (Fig 4B’) compared with controls (Fig 4A’), which suggested that some VG neurons projected to the apex of the OC in *Prickle1*^*C251X/C251X*^ mutants. Third, in the cochlear nuclei, apical afferents (Fig 4D and 4E) formed collaterals from the main branches that passed the basal turn afferents, and projected to the dorsal-most parts of the cochlear nucleus complex, close to the choroid plexus (Fig 4D and 4E, S2 Fig). Some afferents even projected into the vestibular nuclei (Fig 4D and 4E, arrow). It should be noted some of these afferents innervating vestibular nuclei could be VG neurons (Fig 4B’ and 4E). In addition, the OCE also showed an unusual trajectory, turning posteriorly and crossing afferents from apex (Fig 4D and 4E), rather than dorsally towards the afferents from the base (Fig 4C).

**Fig 5:**
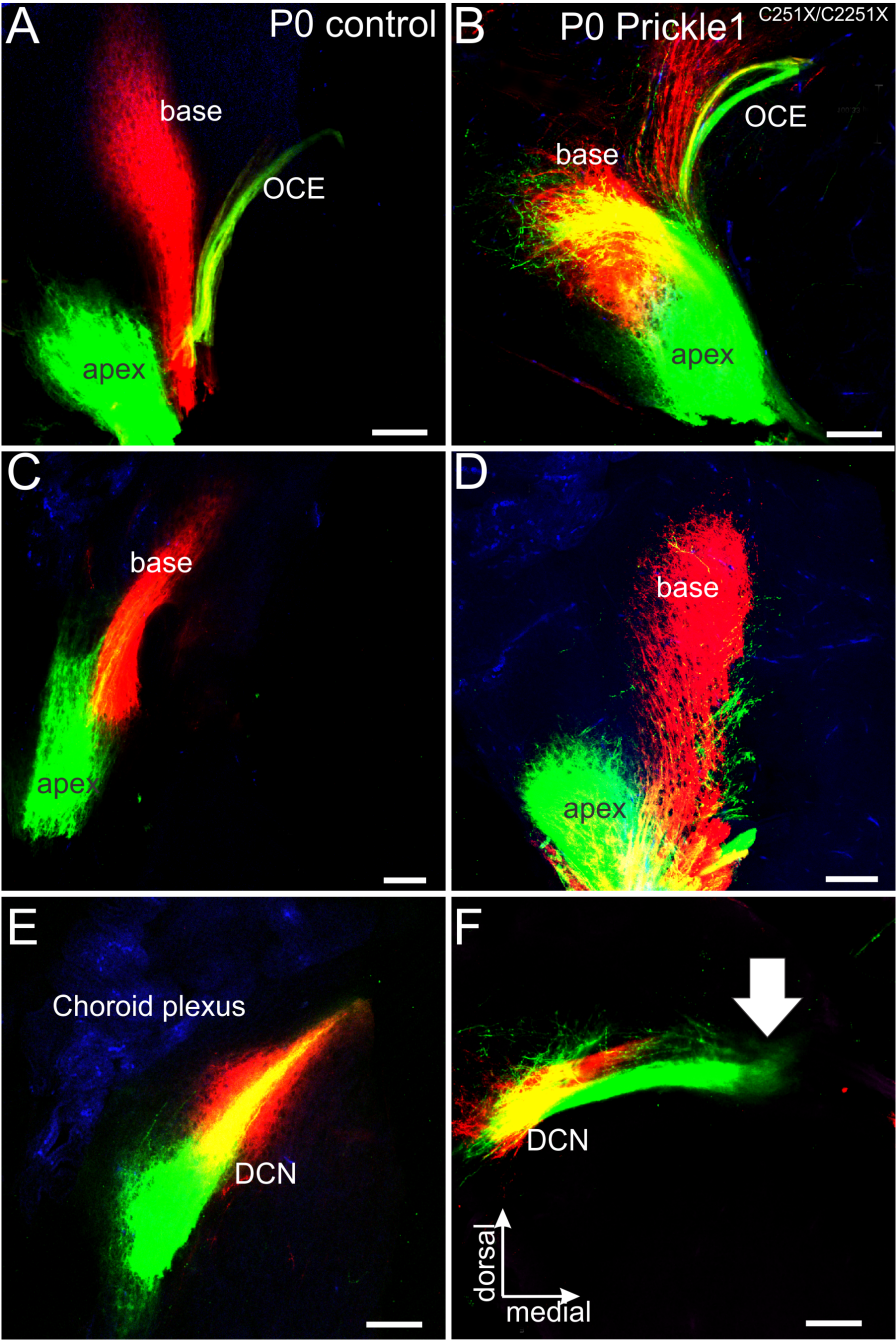
Central projections from apical afferents are expanded in the cochlear nuclei in *Prickle1*^*C251X/C251X*^ mutants. Different colors of lipophilic dye were applied to the apex (green) and the base (red) of the cochlea. The brainstem was sectioned coronally. (A, C, E) In controls, the afferents from base and apex of the cochlea and the olivocochlear efferents (OCE) segregated into bundles and innervate distinct parts of the cochlear nuclei. (B, D, F) In *Prickle1*^*C251X/C251X*^ mutants, the afferents from the apex expanded to the regions where normally basal afferents were. In addition, some apical afferents even projected to the vestibular ganglion (arrow). DCN, dorsal cochlear nuclei. Scale bar, 100 μm.

We also sectioned the cochlear nuclei coronally to analyze in more detail of the overlap of apical afferent with basal afferents (Fig 5). Importantly, this overlap was particularly pronounced near the entry of the cochlear afferents and the OCE (Fig 5A–5D). Again, apical afferents projected collaterals past the cochlear nuclei to vestibular nuclei (Fig 3D and 3E, Fig 5F, arrow).

Our previous work has shown that *Prickle1* mRNA was not detected in the cochlear nuclei from E10.5 to E13.5 by in situ hybridization [36], at which stage the central projections of SGN reach the brainstem. We further analyzed the expression of Prickle1 in the brainstem at later stages by β-Gal staining, to rule out the possibility that the central projection defects in *Prickle1C251X* mutants were due to loss of Prickle1 in the brain (S3 Fig). At P0, there was no Prickle1 expression in the cochlear nucleus (S3A’ Fig). These data together suggest the central projection defects in *Prickle1*C251X mutants were possibly cell-autonomous.

We also analyzed the distal and central projection in E16.5 day old mutant mice, whose apical type II afferent growth had not yet started. We could not detect any type II fibers growing out distally at this stage (data not shown). The central projection of base and apical afferents had already formed at this stage as previously described [42, 46]. These data suggested that expansion of the central projection from apical SGNs coincides with outgrowth of type II afferents to apical outer hair cells from E16.5 to P0.

### Prickle1 is not required for hair cell polarity formation

Prickle1 protein has been shown to be asymmetrically localized in the hair cells [27], and we were able to detect very weak β-gal staining in the OC at P0 (S1 Fig). We therefore analyzed the hair cell polarity in *Prickle1* mutants.

**Fig 6:**
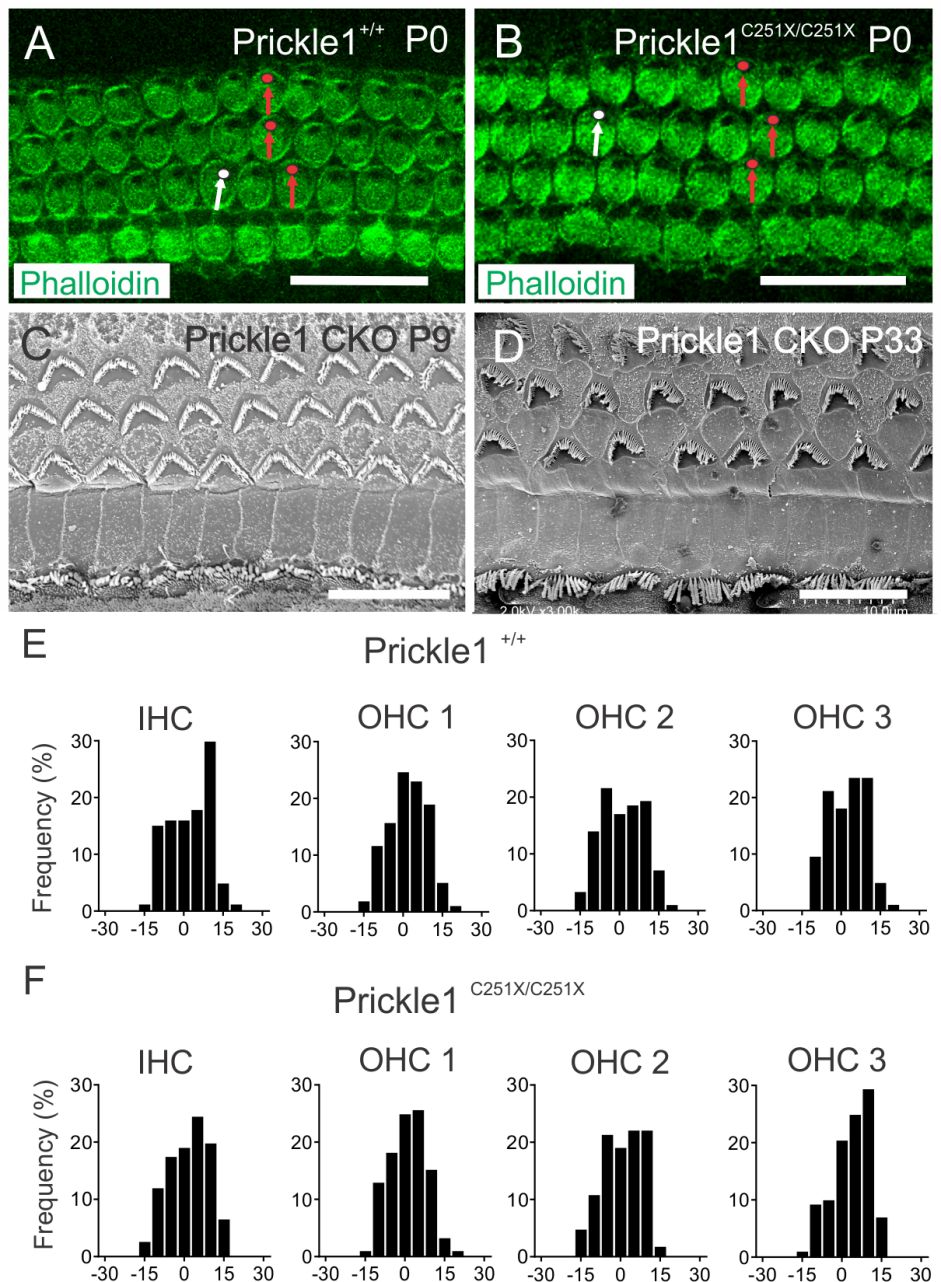
Prickle1 mutation has no discernible effect on hair cell polarity. (A-B) Hair cell polarity was analyzed by the hair cells with phalloidin, which did not stain the fonticulus (black dot within the hair cell surface). At P0, in *Prickle1*^*C251X/C251X*^ mutant (B) compared with *Prickle1*^*+/+*^ (A), most hair cells had fonticulus organized at the lateral side (red arrows and circles). There were a few cells that are slightly mis-oriented in either *Prickle1*^*C251X/C251X*^ or *Prickle1*^*+/+*^ OC (white arrows and white circles). (C-D) Hair cell polarity as analyzed by SEM *Prickle1*^*f/f*^*; Pax2-cre* mutant at P9 (C) or P33 (D). (E-F) The degree of fonticular rotation was quantified at various locations along the cochlea in both Prickle1^+/+^ (E) and *Prickle1*^*C251X/C251X*^ (F) mice at P0, with positive degree meaning rotation towards the apex and negative meaning rotation towards the base. The frequency distribution of the individual row of hair cells was plotted against the degree of rotation. 5 degrees were binned into one bar. About 130 hair cells were analyzed for each row of hair cells. 3 *Prickle1*^*+/+*^ and 3 *Prickle1*^*C251X/C251X*^ mice were analyzed. ANOVA analysis was performed. Scale bar, 10 μm.

We labeled the *Prickle1*^*C251X/C251X*^ hair cells with phalloidin, which labeled the cuticle plate and stereocilia but left the position of the kinocilia, known as the fonticulus, unlabeled (Fig 6A and 6B, red and white circles). We analyzed the cell orientation by examining the position of the fonticulus in relation to the neural-abneural axis of the OC. In agreement with previously reported findings [31], the fonticulus in most of the HCs was organized properly in the abneural side of the cochlea in both *Prickle1*^*+/+*^ and *Prickle1*^*C251X/C251X*^ mice (Fig 6A and 6B, red arrows). Occasionally, there are a few hair cells slightly mis-oriented, but such slight misalignments were observed in both *Prickle1*^*+/+*^ and *Prickle1*^*C251X/C251X*^ mice (Fig 6A and 6B white arrows). No overt mis-orientation was observed as in *Vangl2* mutants [6]. We quantified HC polarity by measuring the angle between the fonticulus and the neural-abneural axis of the OC using ImageJ. We assigned a fonticulus turning towards apex as a positive angle, while one turning towards the base as a negative angle. We plotted the frequency distribution (%) of kinocilia rotation angle against degree of rotation (Fig 6E and 6F), and ANOVA analysis was performed comparing corresponding OHCs and IHCs between *Prickle1*^*+/+*^ (Fig 6E) and *Prickle1*^*C251X/C251X*^ (Fig 6F). There was no difference in rotation of corresponding row of HCs (ANOVA, *p* > 0.8 for each comparison).

*Prickle1*^*LacZ/LacZ*^ mice die around E5.5 to E6.5 [32]. The survival of *Prickle1*^*C251X/C251X*^ mice to birth suggests that the *Prickle1C251X* mutant protein might still be functional in certain processes. Therefore, we analyzed the hair cell polarity in Prickle1^f/f^; Pax2-cre mice, which specifically lacked Prickle1 expression in the inner ear through conditional deletion without side effects of early lethality. We used scanning electron microscopy (SEM) to analyze the hair cell polarity in P9 (Fig 6C) and P33 (Fig 6D) mice, when the HC PCP development was completed. In agreement with *Prickle1C251X* mutant mice, most of the hair cells had their stereocilia localized laterally in the hair cells, indicating no overt mis-orientation of hair cells beyond occasional misalignment also found in control mice. We did not observe shorter and splayed stereocilia as previously reported [31].

Combining our *in situ* hybridization and β-gal data (Fig 1, S1 Fig), Prickle1 was highly expressed in the spiral ganglion, and weakly expressed in the OC. The expression profile was consistent with the spiral ganglion projection phenotype. Due to the redundancy of Prickle genes in mammals, we reasoned the lack of phenotype might be due to compensation by other Prickle genes. Therefore, we analyzed the Prickle expression in the cochlea by compiling RNA-Seq data that were readily available at gEAR (http://gear.igs.umaryland.edu/). We only analyzed data from E16 to P1, since this was when hair cell polarity was developing [53–56]. We searched in the database for multiple genes that had a role in establishing PCP of cochlear HCs, and normalized to the Prickle1 expression in the HCs of the same study (Table 1).

**Table 1:** Normalized gene expression in the cochlea from RNA-seq data in multiple studies.

	E16[56]		E16[55]	P0[56]		P0[55]	P0[54]		P1[56]	
	HC	CD-HC	SE	HC	CD-HC	SE	HC	CD-HC	HC	ENHC
Prickle1	1	6.89	1	1	1.443	1	1	4.83	1	1.66
Prickle2	0.27	0.56	0.38	0.73	0.99	0.66	0.01	0.01	14.176	12.29
Prickle3	0.25	0.25	0.18	0.05	0.25	0.18	0.26	0.70	-	-
Testin	3.40	3.33	1.29	1.02	1.96	1.99	0.93	1.74	2.00	10.98
Vangl1	0.01	0.04	0.68	0.03	0.04	1.43	0.01	0.007	2.688	2.31
Vangl2	0.08	0.06	1.81	0.06	0.31	2.65	1.82	1.12	18.541	26.98
Fzd3	18.27	6.57	0.95	16.80	8.10	1.50	5.82	1.57	38.041	29.63
Fzd6	1.61	1.01	1.23	14.51	0.49	3.43	0.01	0.01	21.859	13.29
Celsr1	0.56	0.27	2.09	0.77	0.26	5.41	14.65	1.22	6.53	5.52
Dvl1	0.41	0.27	1.55	0.25	0.15	0.61	2.14	2.12	0.576	0.82
Dvl2	0.81	1.41	1.03	4.74	9.44	1.02	1.77	3.30	1.99	3.42
Dvl3	0.07	0.04	1.75	0.28	0.48	1.52	3.37	2.47	3.18	3.49

RNA-Seq data from 4 studies were compiled and normalized to Prickle1 expression in the hair cells (or sensory epithelium) of the same study. Only expression level at E16, P0 and P1 was analyzed. HC, hair cells; CD-HC, cochlear duct excluding hair cells; SE, sensory epithelium; ENHC, epithelial non-hair cells.

Based on these studies, we made the following observations: 1) *Prickle1* mRNA was the most abundant Prickle member in the cochlea from E16 to P0; 2) *Prickle1* mRNA was expressed in higher levels in non-hair cells than hair cells; 3) *Prickle1* expression was lower than *Testin* and *Fzd3* in four studies we analyzed; and 4) the relative expression of *Vangl1/2*, *Fzd6*, *Celsr1*, and *Dvl1/2/3* to Prickle1 were different across different studies, which required further analysis.

The higher expression of *Prickle1* in the non-hair cells suggests that Prickle1 could be expressed in the supporting cells, similar to Vangl2 [7] and Prickle2 [18]. It could also be explained by Prickle1 expression in the stria vascularis (Fig 1). Nevertheless, since we only detected weak expression in the OC but strong expression in the SG, the role of Prickle1 in cochlear HC PCP development could be minor. In agreement with this, even though *Testin* and *Fzd3* were more abundant in the OC than *Prickle1*, single loss of these two genes does not cause PCP defects in the cochlear HCs [9, 30].

Prickle2 and 3 are also expressed in the OC, although at much lower levels than Prickle1. Due to this, we cannot rule out the possibility of Prickle2/3 compensating for the loss of Prickle1. Prickle4 is not currently in the database, but since it only has 2 LIM domains [57], it is less likely to compensate for loss of Prickle1. In addition, loss of Prickle2 has no effect on cochlear HC PCP either [58]. Therefore, to analyze the effect of Prickle, it will require knocking out all Prickles, or combining loss of Prickle1 and Vangl2.

We asked if hearing was affected by loss of Prickle1. We tested the hearing threshold with pure tone acoustic brainstem response (ABR) test on *Prickle1*^*f/f*^*; Pax2-cre* mice of P21–23 days old. We tested 3 CKO and 5 control mice, and there was a statistically significant difference between controls and *Prickle1CKO* mice (S4 Fig, 2-way ANOVA, *p* < 0.001). Nevertheless, our data show a prominent role of Prickle1 in the distal and central projection of SGNs, in agreement with the strong expression in the spiral ganglion, rather than regulating HC PCP in the cochlea.

## Discussion

### Prickle1 is not required for the development of hair cell PCP in the cochlea

The function of Prickle1 has been closely linked with Wnt/PCP signaling in the development of several systems, such as limb [34], palate [35] and facial motor neurons [36]. In contrast to these findings, and using the same mutants, we found that in one of the most pronounced displays of PCP—HCs of the organ of Corti, hair cell PCP is not disrupted in *Prickle1*^*C251X/C251X*^, *Prickle1*^*b/b*^, or *Prickle1*^*f/f*^*; Pax2-cre* mutant mice (Fig 6 and [31]). While we found occasional aberrant hair cells, we also found similar aberrant hair cells in control animals. We conclude that this mouse line has an occasional aberration of hair cell development that could relate to some dysregulation of transcription factors during development as previously described [59].

This lack of an obvious phenotype is consistent with the limited, at best, expression of Prickle1 in the OC (Fig 1) and could be due to the redundancy of multiple Prickles in the cochlea, as is the case with the Fzd, Vangl, and Dvl families [9, 10, 14]. For instance, Testin, a protein structurally similar to Prickle1, plays a role in cochlear HC PCP development and genetically interacts with Vangl2 [30]. Therefore, Testin might be the intracellular partner to mediate Vangl2 signaling in the formation of hair cell polarity in the cochlea, although this requires further confirmation that the two protein binds in HCs [30]. However, Prickle2 and Prickle3 are expressed at a much lower level than Prickle1 from E16 to P0 in the OC (Table 1) [53–56, 60], and loss of Prickle2 does not lead to PCP defects in cochlear HCs [58]. These data suggested that Prickle1 was the most important Prickle member in the HC PCP development. We still cannot exclude the possibility of other Prickles compensating for Prickle1 in our mutants, but it requires knocking out multiple Prickles to analyze this possibility.

It is unlikely that the normal hair cell polarity in these *Prickle1* mutant lines is due to incomplete loss of Prickle1 protein function in the hair cells, given the weak expression of Prickle1 in the HCs of the organ of Corti (Fig 1, S1 Fig and Table 1). Supporting this expression pattern, we show a phenotype in *Prickle1* mutant SGNs, but not in hair cells. In addition, we have found morphological defects and early lethality in *Prickle1C251X* mutants [34–36].

### Prickle1 affects neurite growth

In the mouse, Prickle1 has been shown to regulate neuron morphogenesis, including neuron migration [36] and neurite growth [26, 39]. Our work is the first to show Prickle1 is involved in regulating distal and central outgrowth of SGN neurites of the inner ear. In *Prickle1*^*C251X/C251X*^ mutants, the outgrowth of type II SGN neurites in the apex is impaired: there are neurites that turn towards the apex instead of the base, that branch to innervate multiple rows of hair cells, and that fail to project to hair cells (Figs 2 and 3). However, we cannot exclude that the more numerous type I afferents also show unusual branching in the apex. To our knowledge, such unusual branches have not been described before in investigations of normal type II development [47, 48, 61]. A somewhat similar phenotype has previously been described in *Prox1* mutants with a conditional deletion restricted to spiral ganglion neurons that may be increased by the Prox1 expression in supporting cells [62].

Compared with the *Prickle1C251X* mutants, *Prickle1CKO* mice had less severe outgrowth defects (Figs 2 and 3). This could be explained several ways: 1) Prickle1C251X mutant protein, which had the third LIM domain and the C-terminal nuclear localization signals deleted, probably acts as a dominant-negative protein and competes with normal Prickle family function, as is the case with *Vangl2 Lp* mutants [18, 26]; 2) there could be an unknown mechanism that prunes the aberrant fibers during post-natal development; and 3) Pax2-cre only provides a delayed knockout of the gene, compared with Foxg1-cre [63].

Consistent with the unusual branching morphology of some type II afferents in the apex of the cochlea, we also found central projections of apical afferents displaying unusual branching beyond the short collaterals typically found [51, 52, 64]. It is possible that in the absence of Prickle1, apical SGNs adopt a partial vestibular ganglion neuron phenotype (Fig 4B’, 4D and 4E). Nevertheless, this phenotype is clearly different from the only other afferent disorientation cochlear afferent phenotype reported thus far, *Npr2* mutants [64]. Given that branching is clearly more profound in the dorsal cochlear nucleus, it is tempting to speculate that these collaterals are mainly coming from type II afferents known to reach the granular cap [51]. Genetic labeling of only some afferents [48] is needed in the *Prickle1* mutant mice to demonstrate that indeed only a subpopulation of type II fibers responds to loss of Prickle with excessive branching in the cochlea and in cochlear nuclei.

To our knowledge, Prickle1 is the first member in the Wnt/PCP signaling that regulates apical type II fiber branching (but possibly also type I) and direction of growth. However, whether this function of Prickle1 is part of the Wnt signaling is unknown. Wnt signaling can either attract or repel axon outgrowth in the central nervous system using Fzd3/Ceslr3 [65, 66] and both are expressed in developing SGNs (http://www.eurexpress.org/). Several Wnts are expressed in the chicken inner ear, including Wnt5a, and their expression flank the neurons, suggesting a role in axon guidance [67]. However, over-expression or mis-expression of Wnt5a in the chicken inner ear does not affect axon outgrowth [68]. Function of Wnt5a in neurite outgrowth in the mouse remains to be analyzed.

Our results do not support the role of Prickle1 as previously reported [27]: loss of Prickle1 does not cause PCP defects in the cochlear HCs. In this previous study, Prickle1 is abundantly expressed in the cuticle plate, which is not consistent with the localization of other PCP proteins: Vangl, Fzd and Dvl, are enriched in the membrane, and more restricted on one side of cell rather than the diffuse expression along the whole cell membrane. Ours and others’ expression data suggest that Prickle1 is only weakly expressed in the HCs, but probably moderately expressed in the supporting cells, similar to Vangl2 and Prickle2. These data support the current PCP model in which Vangl/Prickle complex and Fzd function across the cell membrane between neighboring cells (HC-SC boundary), rather than within the same cell (HCs). Moreover, the mechanism of asymmetric, Smurf mediated Prickle1 localization proposed by Narimatsu, et. al. [27] cannot be easily integrated into the emerging model of HC PCP organization [21, 69]: the PCP signaling is only synchronizing the asymmetric movement of the kinocilium across all HCs of the entire epithelium, which could also be compensated by other unknown mechanism [8].

In conclusion, our work shows that Prickle1 is highly expressed in the spiral ganglion and regulates the distal and central outgrowth of the neuronal processes. In contrast to some other studies, we only detected a weak signal in the organ of Corti (possibly in non-hair cells), and we did not find HC polarity deficits in the OC. These results are in agreement with a previous study that analyzed a different *Prickle1*^*-/-*^ mutant [31] but contrast with claims based on immunocytochemistry. Although the role of Prickle1 in HC polarity appears to be insignificant based on our findings, our data also support an interesting and as-of-yet undiscovered role for Prickle1 in the proper projection and branching only of the apical SGNs, who may have a unique evolutionary history [70]. This further adds to the discussion of the role that PCP-related proteins play in the development of hearing.

## Supporting information

S1 Fig Prickle1 is weakly expressed in the organ of Corti but strongly expressed in the spiral ganglion. Cochleae from *Prickle1*^*LacZ/+*^ mice of P0 (A) or P6 (B) were stained with β-Gal. OC, organ of Corti; SG, spiral ganglion. Scale bar, 100 μm.
(TIF)

S2 Fig Central projections from apical afferents are expanded in the cochlear nuclei in *Prickle1^C251X/C251X^* mutants. (A) Lipophilic dye was applied to the apex of a mutant cochlea. Blue, auto-fluorescence. (B) A subset of olivocochlear efferents (OCE) failed to form a nice bundle as they were passing the vestibular ganglion. (C) The afferent from apical cochlea separated and projected to almost the whole entire cochlear nuclei. Inset, a lower magnification view of C showing choroid plexus. AVCN, anterior-ventral cochlear nuclei; DCN, dorsal cochlear nuclei. Scale bar, 100 μm.
(TIF)

S3 Fig Prickle1 is not expressed in the cochlear nucleus. Brain from *Prickle1*^*LacZ/+*^ mice of P0 (A, A’) or P10 (B, B’) were sectioned at the mid-sagittal plane, and stained with β-Gal. The staining was shown from the medial side (A, B) and the lateral side (A’, B’). CP, choroid plexus; RF, reticular formation; IO, inferior olivary complex; CN, cochlear nucleus; CB, cerebellum.
(TIF)

S4 Fig Hearing threshold is impaired in *Prickle1 CKO* mice at P21-P23. Hearing threshold from *Prickle1*^*f/f*^*; pax2-cr*e and control mice were analyzed at 8, 16, and 32 kHz using pure tone ABR test. 2-way ANOVA (genotype, *p* < 0.001) and post-hoc Bonferroni’s multiple comparisons test was performed: *, *p* < 0.05. Five controls and three mutants were analyzed.
(TIF)

S1 File The ARRIVE guidelines checklist for reporting *in vivo* experiments. See the checklist and text for details.
(PDF)
