# Supplementary figures and images for: Prickle1 regulates neurite outgrowth of apical spiral ganglion neurons but not hair cell polarity in the murine cochlea
Source: PLoS One. 2017 Aug 24;12(8):e0183773. doi: 10.1371/journal.pone.0183773 (PMC5570324; doi:10.1371/journal.pone.0183773)

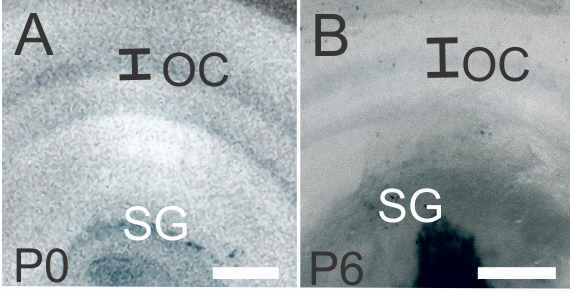

Supplement: S1 Fig — Cochleae from Prickle1LacZ/+ mice of P0 (A) or P6 (B) were stained with β-Gal. OC, organ of Corti; SG, spiral ganglion. Scale bar, 100 μm. (TIF) [file pone.0183773.s001.tif]

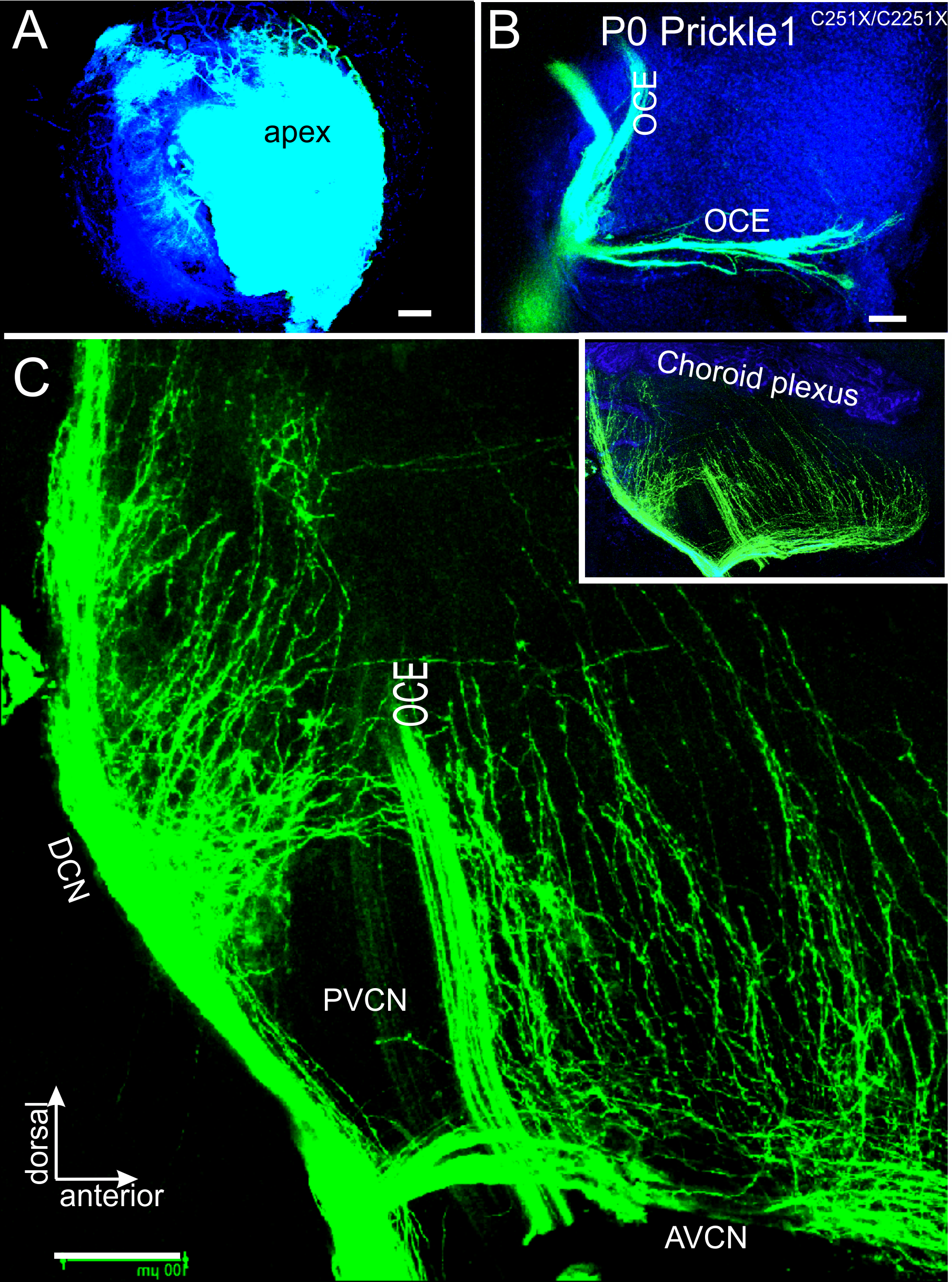

Supplement: S2 Fig — (A) Lipophilic dye was applied to the apex of a mutant cochlea. Blue, auto-fluorescence. (B) A subset of olivocochlear efferents (OCE) failed to form a nice bundle as they were passing the vestibular ganglion. (C) The afferent from apical cochlea separated and projected to almost the whole entire cochlear nuclei. Inset, a lower magnification view of C showing choroid plexus. AVCN, anterior-ventral cochlear nuclei; DCN, dorsal cochlear nuclei. Scale bar, 100 μm. (TIF) [file pone.0183773.s002.tif]

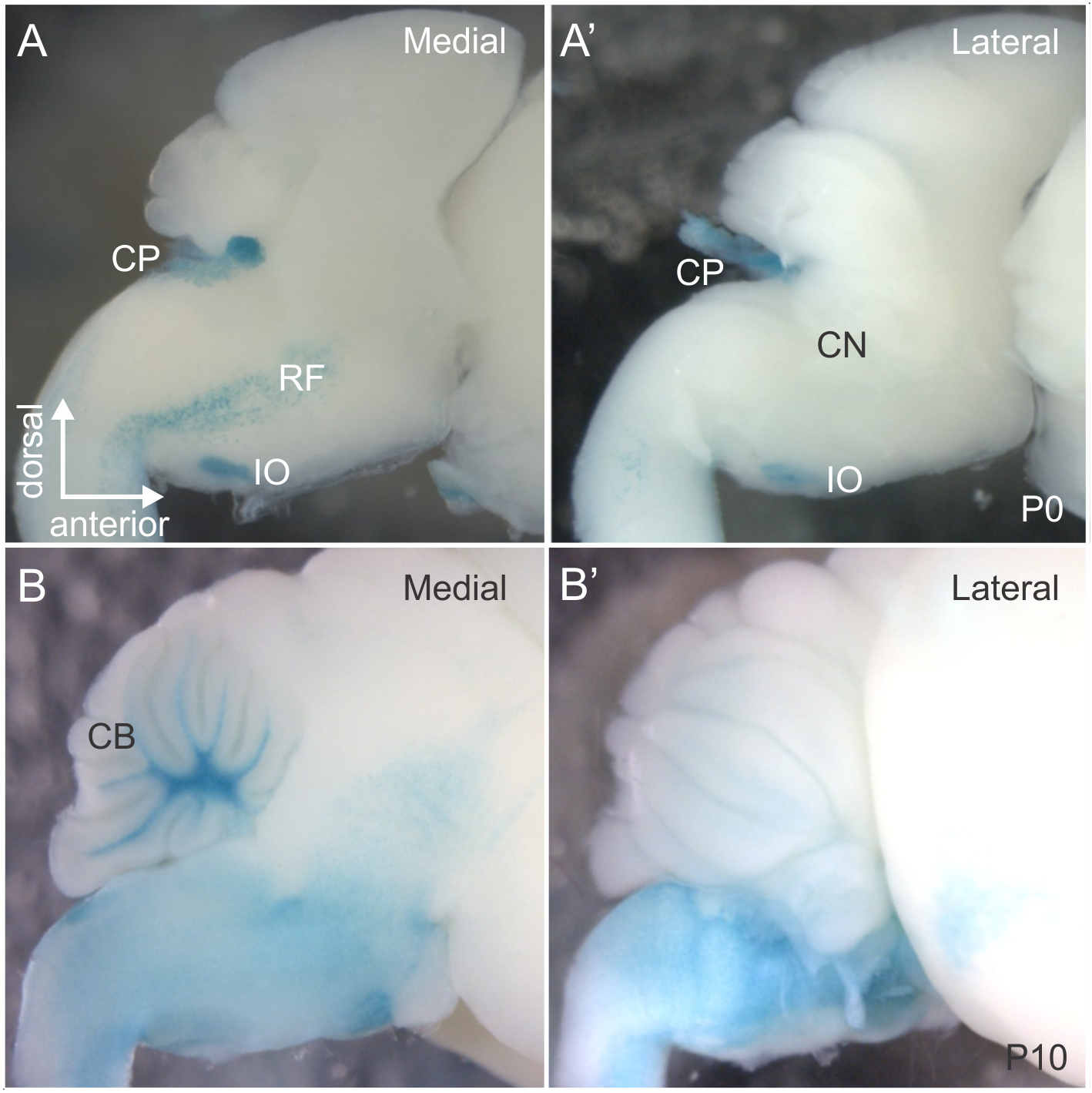

Supplement: S3 Fig — Brain from Prickle1LacZ/+ mice of P0 (A, A’) or P10 (B, B’) were sectioned at the mid-sagittal plane, and stained with β-Gal. The staining was shown from the medial side (A, B) and the lateral side (A’, B’). CP, choroid plexus; RF, reticular formation; IO, inferior olivary complex; CN, cochlear nucleus; CB, cerebellum. (TIF) [file pone.0183773.s003.tif]

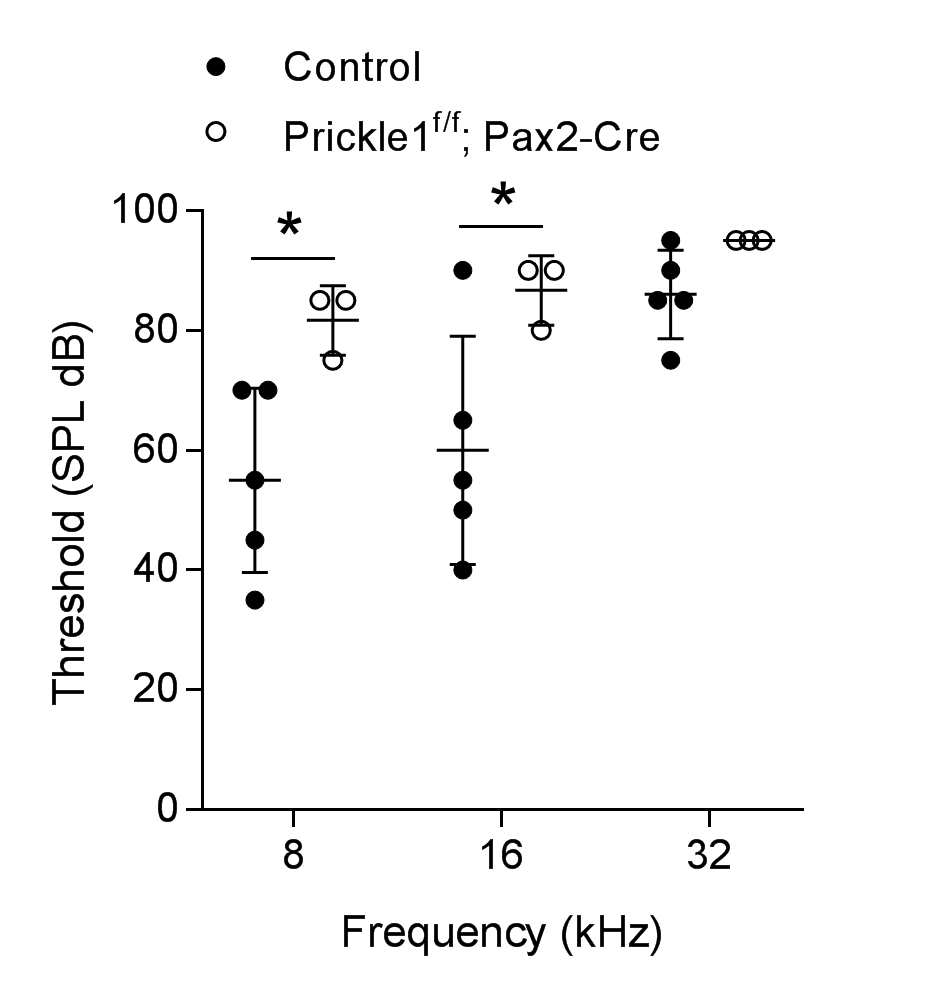

Supplement: S4 Fig — Hearing threshold from Prickle1f/f; pax2-cre and control mice were analyzed at 8, 16, and 32 kHz using pure tone ABR test. 2-way ANOVA (genotype, p < 0.001) and post-hoc Bonferroni’s multiple comparisons test was performed: *, p < 0.05. Five controls and three mutants were analyzed. (TIF) [file pone.0183773.s004.tif]
